# The irritant receptor TRPA1 mediates the mosquito repellent effect of catnip

**DOI:** 10.1016/j.cub.2021.02.010

**Published:** 2021-03-04

**Authors:** Nadia Melo, Matthew Capek, Oscar M. Arenas, Ali Afify, Ayse Yilmaz, Christopher J. Potter, Peter J. Laminette, Alessia Para, Marco Gallio, Marcus C. Stensmyr

**Affiliations:** 1 Department of Biology, Lund University, 22362 Lund, Sweden; 2 The Solomon H. Snyder Department of Neuroscience, Johns Hopkins University School of Medicine, Baltimore, MD 21205, USA; 3 Department of Neurobiology, Northwestern University, Evanston, IL 60208, USA

## Abstract

Catnip (*Nepeta cataria*) is a common garden herb well known for its euphoric and hallucinogenic effects on domestic cats,^1–3^ for its medicinal properties,^4,5^ as well as for its powerful repellent action on insects.^6,7^ Catnip extracts have been proposed as a natural alternative to synthetic insect repellents, such as N,N-diethyl-3-methylbenzamide (DEET),^8,9^ but how catnip triggers aversion in insects is not known. Here, we show that, both in *Drosophila melanogaster* flies and *Aedes aegypti* mosquitoes, the major mediator of catnip repellency is the widely conserved chemical irritant receptor TRPA1. *In vitro*, both catnip extract and its active ingredient nepetalactone can directly activate fly and mosquito TRPA1. *In vivo*, *D. melanogaster* and *Ae. aegypti TRPA1* mutants are no longer repelled by catnip and nepetalactone. Interestingly, our data show that some, but not all, fly and mosquito TRPA1 variants are catnip targets. Moreover, unlike the broad TRPA1 agonist allyl isothiocyanate (AITC) (an active ingredient of tear gas and wasabi), catnip does not activate human TRPA1. Our results support the use of catnip and nepetalactone as insect-selective irritants and suggest that, despite TRPA1’s broad conservation, insect TRPA1 can be targeted for the development of safe repellents.

## In brief

Catnip has been used for millennia as an insect repellent. Melo et al. find that catnip and its major iridoid component nepetalactone activate insect isoforms of the irritant receptor TRPA1. Mosquitoes lacking TRPA1 are no longer repelled by catnip. Catnip does not activate human TRPA1, and this supports its use as a safe natural mosquito repellent.

## Graphical Abstract

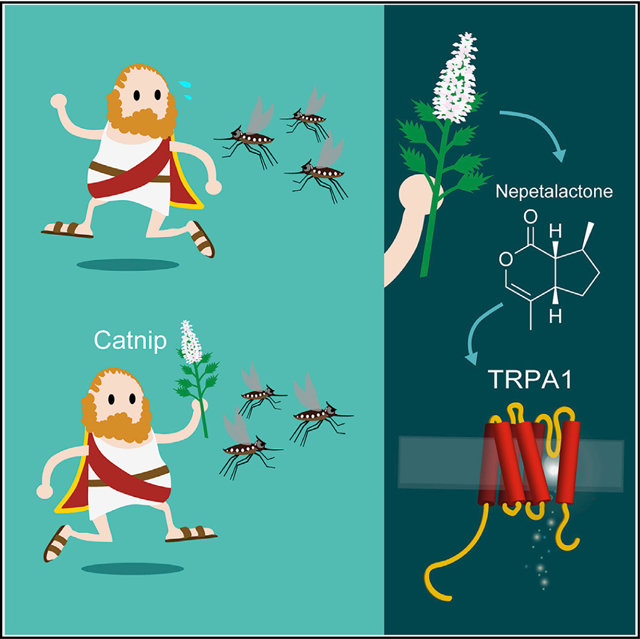

## RESULTS AND DISCUSSION

**Figure 1.**
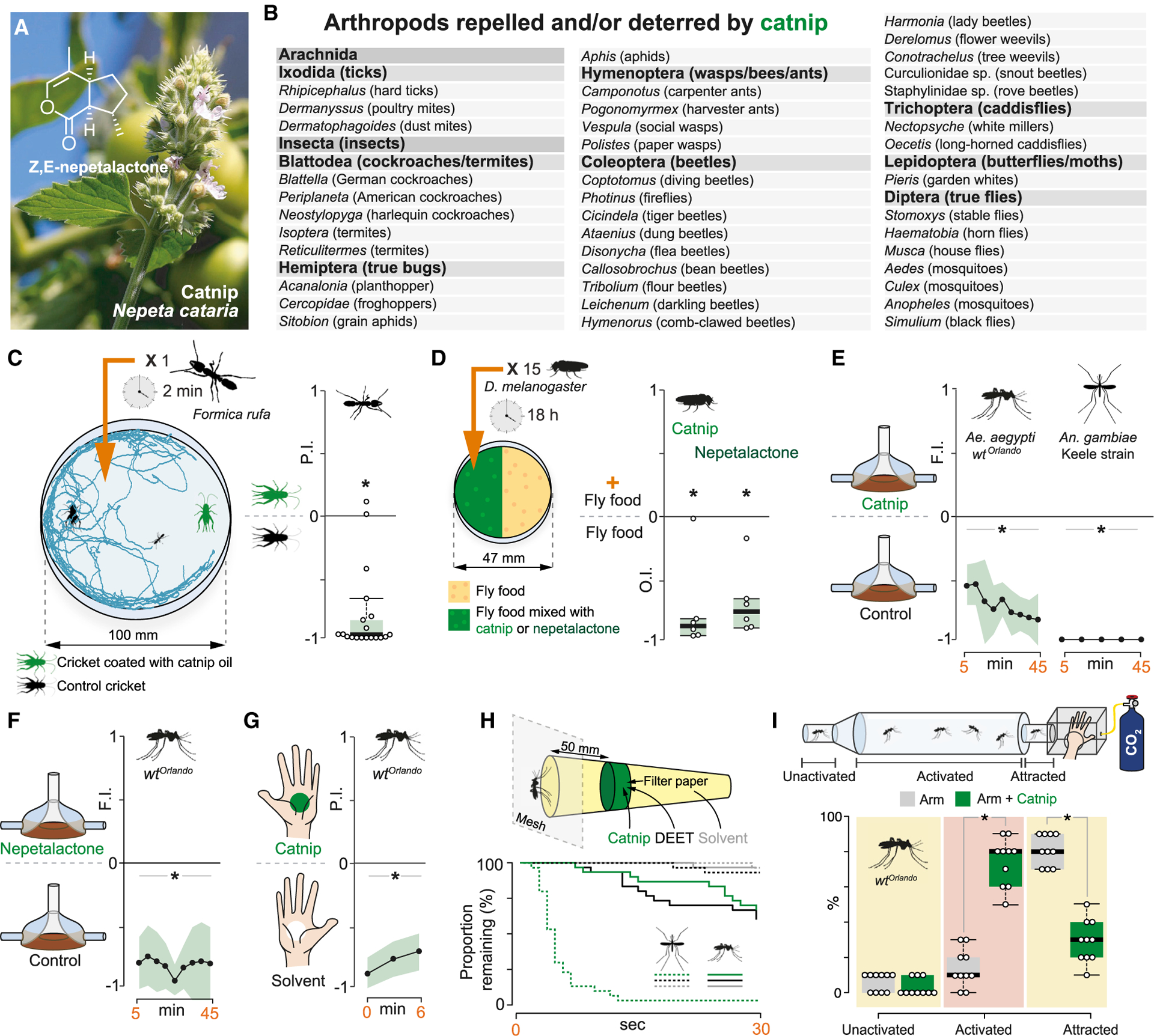
Catnip is aversive to a wide range of arthropods (A) Catnip (*Nepeta cataria*) and the dominant Z,E-nepetalactone isomer that makes up most of the volatile headspace. Photo: M. Stensmyr. (B) Arthropods shown to be repelled by catnip and/or nepetalactone. See extended Table S1. (C) Schematic of the ant behavioral assay, with a representative track of an ant choosing between a cricket coated with catnip extract (5 μL) and a control cricket. Right: quantified behavior shows time spent near the catnip-treated or the control cricket (1 ant/trial; n = 20). The edges of the boxes are the first and third quartiles, thick lines mark the medians, and whiskers represent data range. Preference was tested with one-sample Wilcoxon test, theoretical mean 0. Star denotes significantly different from 0; p < 0.05. (D) Schematic of the *Drosophila melanogaster* oviposition assay. Oviposition indices (OIs) of flies provided a choice to lay eggs on standard cornmeal or fly food with either catnip (0.05 g) or nepetalactone (150 μL 10^−6^) added. Boxplots and statistics are as per (C). (E) Feeding indices (FIs) over 45 min from *Ae. aegypti* (30 mosquitoes/trial; n = 6) and *An. gambiae* provided a choice to feed from two membrane blood feeders, one of which treated with catnip (1 g). Shaded line indicates SEM. Preference was tested with one-sample Wilcoxon test, theoretical mean 0, for each time point. (F) FI over 45 min from *Ae. aegypti* (30 mosquitoes/trial; n = 6) given a choice to feed from two membrane blood feeders, one of which treated with nepetalactone (150 μL). Shaded line indicates SEM. Preference was tested with one-sample Wilcoxon test, theoretical mean 0, for each time point. (G) Probing index (PI) over 6 min from wild-type (WT) *Ae. aegypti* (20 mosquitoes/trial; n = 6) in a modified constrained feeding access assay, provided with a choice to approach and probe two hands (from the same individual), one of which scented with catnip. Shaded line indicates SEM. Statistics are as per (C). (H) Kaplan-Meier estimates showing the proportions of *Ae. aegypti* and *An. coluzzii* mosquitoes remaining on the cage wall over time in response to catnip, DEET, and the paraffin oil control (n = 30 mosquitoes for each species). Stars indicate significant differences from control (Cox proportional hazard model; p < 0.05). (I) Uniport assay trials with *Ae. aegypti* (10 mosquitoes/trial; n = 10) freely orienting in a CO_2_ spiked airstream toward an exposed human arm (with or without catnip oil). Boxplots are as per (C). Statistical difference was measured via a Student’s t test. Star denotes significant difference (p < 0.05). See also Table S1.

The Eurasian herb catnip (*Nepeta cataria*; Figure 1A) is well known for its euphoric and hallucinogenic effects on cats.^1–3^ This plant has, moreover, a long history of use in herbal medicine, possibly dating back to the late Neolithic.^10^ Pliny the elder (AD 23[24]–AD 79) mentions many medicinal uses of *Nepeta* in his *Naturalis Historia*,^4^ and the 9^th^-century *Bald’s Leechbook* reports catnip as effective against everything from bedevilment (mix leaves with ale, chant 12 masses) to shoulder pain (pound leaves in ale, drink by fire).^5^ Although many, or most, of these claimed curative properties remain to be verified (or refuted), catnip does seem to have a positive influence on human well-being, producing a soothing and calming sensation.^11^ To invertebrates, however, catnip is likely to appear less benign, as many insect taxa react with avoidance when confronted with its extracts (Figure 1B; Table S1). This well-known property of catnip explains the long history of its use as an insect repellent. For example, in Johannes Franck’s *Speculum botanicum* from 1638,^6^ the first comprehensive botanical work from Sweden, catnip is mentioned as *lössegräs,* a name also later used by Carl von Linné.^7^ *Lössegräs* can be loosely translated into “lice grass” (where “lice” likely refers to all pesky small creatures), alluding to its use as an insect repellent.

Although likely in use for millennia to ward off troublesome invertebrates, catnip has only more recently been systematically explored as a potential insect repellent, heralded as a natural alternative to the synthetic chemicals dominating the market. Many studies have reported promising results (Table S1), with catnip even comparing favorably to the present gold-standard mosquito repellent DEET (N,N-diethyl-3-methylbenzamide) in some studies.^8,9^ The effect of catnip on both cats and insects has been attributed to a class of structurally closely related iridoids, a type of monoterpenoid, and in particular to two isomers of nepetalactone (E,Z and Z,E), which typically make up to 80% of catnip extracts (Figure 1A).^2,12^ The catnip iridoids have been suggested to activate the cat μ-opioid pathway, thereby triggering the brain reward systems;^3,13^ in contrast, why insects are strongly repelled by catnip remains unknown.

### Catnip is a broad-acting insect repellent

To begin investigating the molecular mechanism of catnip action, we established a series of laboratory assays to quantify its repellency on invertebrates. We first revisited a claim attributed to Pliny, namely that catnip repels scorpions. We allowed individual scorpions (*Heterometrus cyaneus*) to choose between two pots serving as hiding places, one of which contained catnip. The scorpions (n = 4) all chose the pot *with* catnip, displaying no apparent distress (data not shown). In this instance, we conclude that Pliny may have been misinformed (but note that the plant Pliny calls *Nepeta* might be a completely different herb).^14^ Next, we repeated a more-recent catnip experiment performed on ants by Thomas Eisner in 1964.^15^ We introduced single *Formica rufa* ants into an open arena and presented them with the choice of two dead house crickets (*Acheta domesticus*), one of which had been coated with catnip extract. The ants’ exploratory behavior (recorded for 1 min following introduction into the chamber) confirmed that ants indeed strongly avoid the catnip-laced cricket (Figure 1C). As ants and scorpions provide limited experimental access, we next tested the effect of catnip on the common dew fly *Drosophila melanogaster* using a well-established egg laying assay.^16^ Here too, catnip acted as an aversive agent, as did a racemic mix of E,Z- and Z,E-nepetalactone, the major iridoid components of catnip (Figure 1D), henceforth referred to as just nepetalactone. We next assessed the effect of catnip on the blood-feeding behavior of yellow fever (*Aedes aegypti*) and malaria mosquitoes (*Anopheles gambiae*). Mosquitoes were provided a choice between two membrane feeders covered with worn nylon socks, one of which had been treated with catnip extract. Our results demonstrate that both species strongly avoided drawing blood from feeders covered with catnip-treated socks (Figure 1E; note that, as expected, mosquitoes also avoided feeders treated with nepetalactone; Figure 1F). To further assess the effect of catnip on mosquito behavior, we then tested catnip in a modified constrained feeding access assay.^17^ *Aedes* mosquitoes showed robust aversion toward a human hand coated with catnip (Figure 1G). Together, these results confirm that catnip (and nepetalactone) indeed function as feeding deterrents for blood-feeding mosquitoes.

Catnip has also been suggested to function as a spatial repellent. To test this notion, we first assessed the effect of catnip in a close proximity response assay, where mosquitoes are in close range of the stimulus, but not in direct contact.^18^ In this assay, both *Ae. aegypti* and *Anopheles coluzzii* mosquitoes displayed aversion, but the magnitude of the effect on the two species was different, as *Anopheles* reacted both more strongly and rapidly to the presence of catnip (Figure 1H). In a uniport olfactometer assay,^19^ however, *Aedes* mosquitoes were robustly repelled by catnip at a close distance and showed reduced upwind flight toward an exposed human arm treated with catnip (Figure 1I). In summary, our results confirm that catnip can be regarded as a repellent as well as a feeding deterrent for both *Aedes* and *Anopheles* mosquitoes. The effect of catnip does not require direct contact, because close range is sufficient to induce aversive behavior. Our data also confirm that catnip is aversive to both *Formica* ants and *Drosophila* flies in behavioral assays designed to replicate potentially relevant exposure in these species (foraging and egg laying, respectively).

### Catnip oil and nepetalactone activate insect TRPA1 *in vitro*

How can catnip (and nepetalactone) repel such a broad range of insect species? We reasoned that, given the diverse taxa involved (Figure 1B), the relevant catnip receptor is unlikely to be a specific olfactory channel tuned to iridoids. The fact that catnip can function at a distance also seems to rule out a contact-dependent mechanism, such as the taste receptors for bitter compounds. Instead, the widely conserved irritant receptor TRPA1 could be a good candidate to mediate the broad insect aversion to catnip.

TRPA1 is rather broadly expressed in the insect nervous system,^20–31^ and TRPA1 activation by noxious heat/reactive oxygen species^32^ and irritant chemicals^23^ rapidly triggers robust escape responses. Moreover, small reactive molecules and plant essential oils can diffuse through the insect cuticle (see, e.g., Tak and Isman^33^), potentially reaching TRPA1-expressing somatosensory neurons to mediate aversive responses. Finally, at least one widely used plant-derived mosquito repellent—citronellal—has been shown to function through the activation of TRPA1.^25^

**Figure 2.**
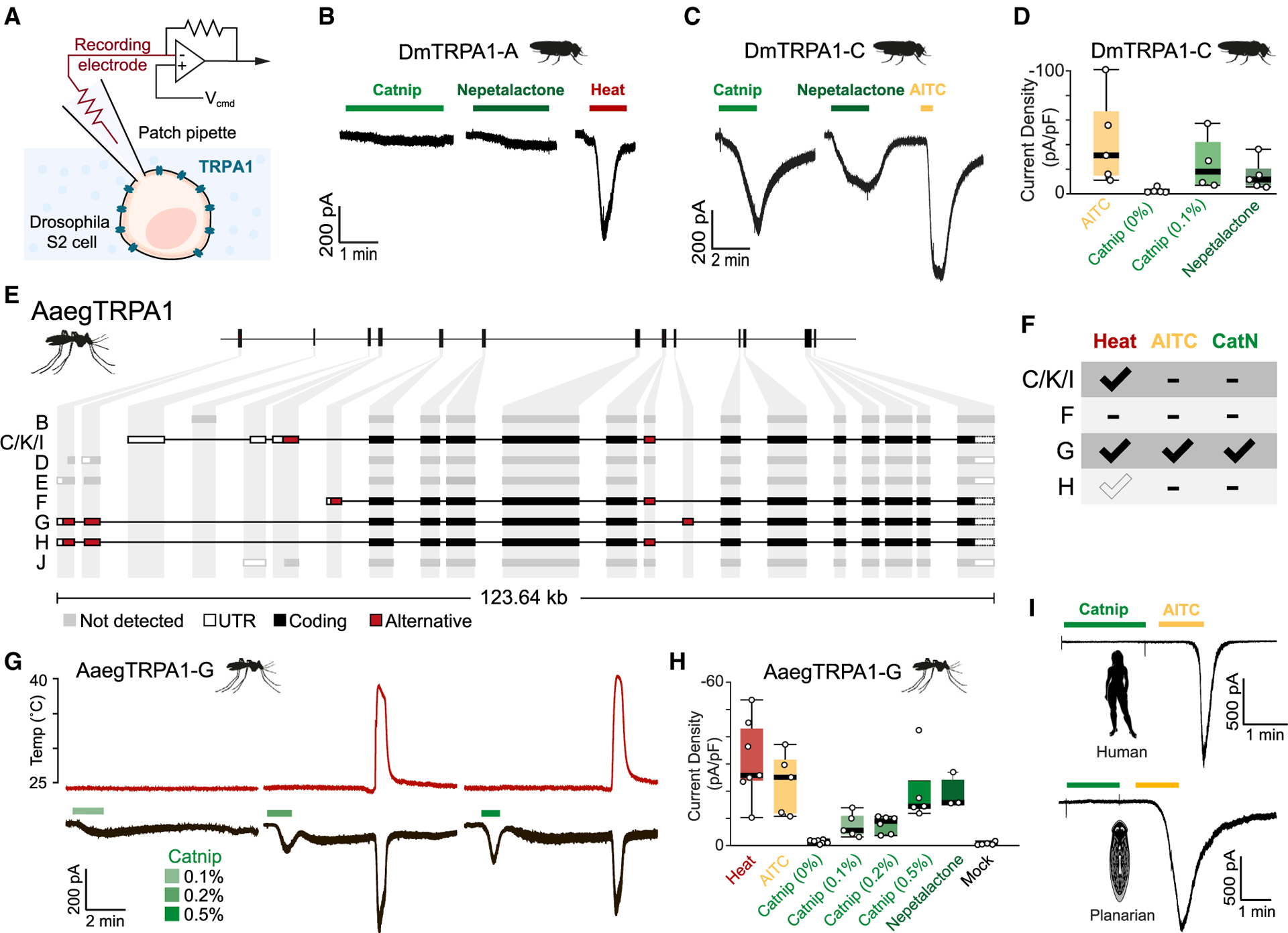
Catnip and nepetalactone activate insect TRPA1 variants *in vitro* (A) Schematic of whole-cell patch-clamp recordings from *Drosophila* S2R+ cells expressing TRPA1. (B and C) Representative current traces from S2R+ cells expressing DmTRPA1 variants during stimulation. (B) DmTRPA1-A-expressing cells stimulated with catnip (0.1%), nepetalactone (100 μM), and heat (32°C). (C) DmTRPA1-C-expressing cells stimulated with catnip (0.1%), nepetalactone (100 μM), and AITC (100 mM). (D) Quantification of responses of DmTRPA1-C-expressing cells to AITC (100 mM), solvent (0% catnip), catnip (0.1%), and nepetalactone (100 μM). Dots represent max current density. The edges of the boxes are the first and third quartiles, thick lines mark the medians, and whiskers represent data range. (E) The *TRPA1* locus in *Aedes aegypti* with 10 predicted transcripts (light gray); the full-length cDNAs cloned here are represented in black and differentially utilized coding exons in red. (F) Summary of the responses of each cloned variant (expressed in S2R+ cells) to stimulation with catnip (0.5%), nepetalactone (100 μM), and heat (32°C). Note that AaegTRPA1-H only weakly responded to heat. (G) Representative current traces from S2R+ cells expressing AaegTRPA1 variants during stimulation with heat and catnip oil (black traces). The red trace above is the temperature recorded in the bath. (H) Quantification of responses of AaegTRPA1-G-expressing cells to heat (32°C), AITC (100 mM), solvent (0% catnip), catnip (0.1%, 0.2%, and 0.5%), and nepetalactone (100 μM). The last column is from mock-transfected cells stimulated with 0.5% catnip. Dots represent max current density. The edges of the boxes are the first and third quartiles, thick lines mark the medians, and whiskers represent data range. (I) Representative current trace from S2R+ cells expressing HsTRPA1 or SmedTRPA1 during stimulation with catnip oil (0.1%) and AITC (100 mM).

To test the possibility that catnip may directly activate insect TRPA1, we first used the well-characterized *D. melanogaster* ortholog, DmTRPA1. Insect TRPA1s have complex loci, often producing different coding variants both from the use of different ATG start codons, as well as from alternative splicing. At least four distinct protein isoforms of TRPA1 are found in flies.^23,34^ Here, we used two representative isoforms that have been previously well characterized *in vitro*:^34^ the heat-sensitive TRPA1A and the heat-insensitive TRPA1C (following the nomenclature in Zhong et al.^34^). These two variants differ in the sequence of amino acids at the N terminus and in ~30 amino acids separating the cytoplasmic ankyrin repeats from the region containing the transmembrane domains.^34^ For this experiment, we expressed each TRPA1 variant in cultured *Drosophila* S2 cells and performed whole-cell patch-clamp electrophysiology (Figure 2A) to measure potential catnip and nepetalactone-induced currents. Our recordings show that, in S2 cells, DmTRPA1C, but not DmTRPA1A, was readily activated by both catnip and nepetalactone (Figures 2B–2D). As a control—and under the same conditions—both isoforms were activated by the broad TRPA1 antagonist allyl isothiocyanate (AITC), and DmTRPA1A was also activated by heat (Figures 2B–2D; data not shown).

Next, we set out to test mosquito TRPA1. A number of distinct TRPA1 variants have been recently described from four disease vector mosquito species (*An. gambiae*, *Anopheles stephensi*, *Ae. aegypti*, and *Culex pipiens pallens*).^35^ The *Ae. aegypti* TRPA1 genomic locus^36^ is predicted to encode at least 10 different transcripts (AAEL001268RB-K; Figure 2E), producing at least 8 different protein variants (AaegTRPA1B-K). As in *Drosophila*, salient differences include alternative N-terminal domains and alternative usage of an internal exon. Starting from an adult cDNA library, we cloned 4 distinct full-length transcripts, corresponding to 4 of the 8 predicted protein variants, and tested them *in vitro* (Figures 2F–2H; see STAR methods for details). When heterologously expressed in S2 cells, only one of the AaegTRPA1-variants (AaegTRPA1G) readily responded to catnip oil and nepetalactone (Figure 2G). AaegTRPA1G responded to catnip in a dose-dependent fashion and was, in addition, activated by heat, as well as by AITC (Figures 2G and 2H). Interestingly, at least one additional protein variant (AaegTRPA1C/K/I) responded to heat, but in this case, we observed no robust activation by AITC. We note that two additional variants (AaegTRPA1F and H) also failed to respond to either catnip or AITC, but in the absence of strong responses to heat, this result has to be taken with caution, as it may reflect poor functional expression of the channel (we note that a previously published *An. gambiae* TRPA1 cDNA^37^ did not produce functional expression in our cell system).

We conclude that some—but not all—*D. melanogaster* and *Ae. aegypti* TRPA1 variants are activated by catnip and nepetalactone. The selective activation of some insect TRPA1 variants may explain the fact that catnip is aversive to some, but not all, insect species. Moreover, as previously mentioned, catnip is not generally aversive outside of the insects; consistently, human TRPA1 was not activated by catnip (Figure 2I), nor was a planarian ortholog (*SmedTRPA1* from *Schmidtea mediterranea*; Figure 2I).

### TRPA1-deficient mosquitoes and flies show no aversion to catnip

**Figure 3.**
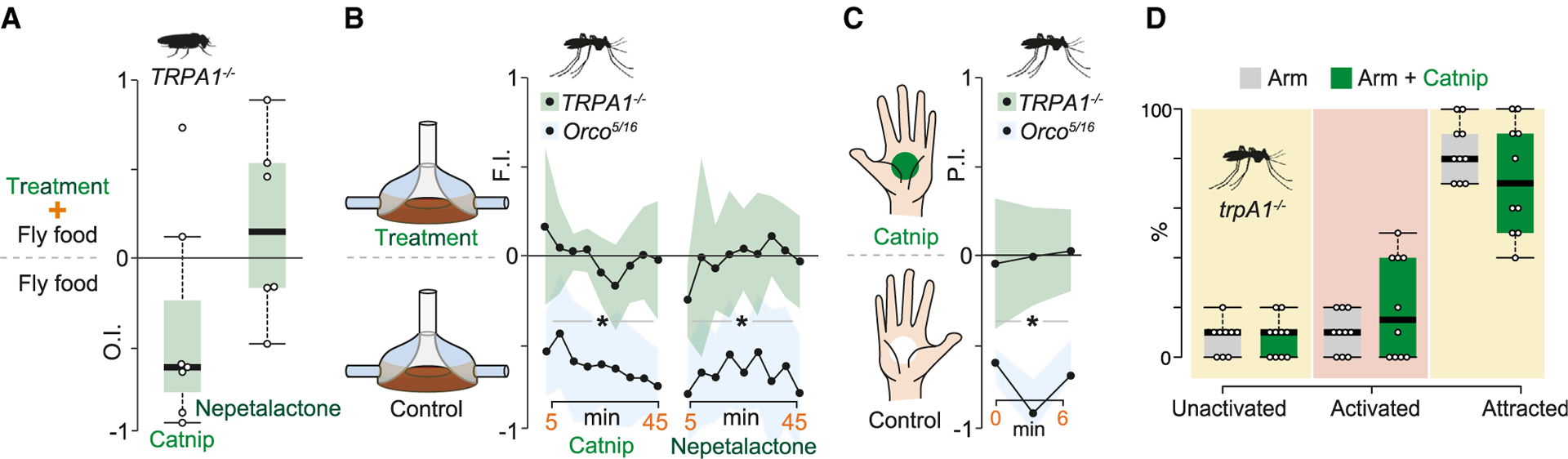
TRPA1 mutant mosquitoes and flies show no aversion to catnip (A) OIs of TRPA1 mutant flies provided a choice to lay eggs on standard cornmeal or fly food with either catnip (0.05 g) or nepetalactone (150 μL 10^−6^) added. Boxplots and statistics are as per Figure 1C. (B) FIs over 45 min from TRPA1 and Orco mutant *Ae. aegypti* (each genotype: 30 mosquitoes/trial; n = 6) provided a choice to feed from two membrane blood feeders, one of which treated with catnip (1 g) or nepetalactone (150 μL 10^−6^). Plots and statistics are as per Figure 1E. (C) PI over 6 min from TRPA1 and Orco mutant *Ae. aegypti* (each genotype: 20 mosquitoes/trial; n = 6) in a modified constrained feeding access assay, provided with a choice to approach and probe two hands (from the same individual), one of which treated with catnip. Shaded line indicates SEM. Statistics are as per Figure 1C. (D) Uniport assay trials with TRPA1 mutant *Ae. aegypti* (10 mosquitoes/trial; n = 10) freely orienting in a CO_2_ spiked airstream toward an exposed human arm (with or without catnip oil). Boxplots are as per (C). Statistical difference was measured via a Student’s t test. No significant difference (p < 0.05).

Is the selective activation of TRPA1 sufficient to explain the strong avoidance to catnip observed in *D. melanogaster* and *Ae. aegypti*? To test this notion, we first performed egg-laying assays on *DmTRPA1* mutant *D. melanogaster*.^38^ Our results show that *DmTRPA1* mutant flies display reduced—but not abolished—aversion to catnip (Figure 3A and compare to Figure 1D). Remarkably, the mutants appear indifferent to nepetalactone and freely laid eggs on the nepetalactone-containing agar, a substrate that is all but avoided by wild-type controls (Figure 3A). These results suggest that catnip extract contains additional components apart from the iridoids that are repellent to *Drosophila* (perhaps activating aversive olfactory channels) but that the avoidance of nepetalactone can be accounted for by DmTRPA1.

We next turned to *Ae. aegypti*, where an *AaegTRPA1* mutant has been recently published,^39^ thus making it possible for us to directly test whether mosquito aversion to catnip could be—partially or fully—explained by the activation of the mosquito TRPA1 channel. Here, we tested *AaegTRPA1* mutant *Ae. aegypti* in three of the previously used assays: the two-choice blood-feeding assay; the modified constrained feeding access assay; and the uniport olfactometer assay. Strikingly, mosquito *AaegTRPA1* mutants displayed no catnip aversion in any of these behavioral assays. In the two-choice blood-feeding assay, *AaegTRPA1* mutant mosquitoes seemed indifferent to catnip (as well as to nepetalactone; Figure 3B), treatments that had been avoided by wild-type controls (see Figure 1E). In the constrained feeding access assay, *AaegTRPA1* mutant *Aedes* readily approached a catnip-laced human hand (Figure 3C), which wild-type animals had nearly completely avoided (Figure 1G). As an additional control, we also tested a different *Ae. aegypti* mutant (an *Orco* mutant,^40^ in which all canonical odorant receptors are rendered nonfunctional,^41^ produced in the same genetic background),^39^ which responded normally to catnip in these assays (Figures 3B and 3C). Thus, even though catnip and nepetalactone activate mosquito olfactory sensory neurons,^42,43^ *Orco*-mediated olfactory input appears to play little role in mediating the aversion toward the iridoids, at least in the assays employed here. Finally, in the uniport olfactometer assay—unlike wild-type controls—*AaegTRPA1* mutant mosquitoes readily approached an exposed human arm laced with catnip (Figure 3D). In summary, these results suggest that catnip and iridoid repellency in *Aedes* mosquitoes can be explained by its ability to activate TRPA1. Iridoid aversion accordingly differs from that elicited by, e.g., DEET, where multiple pathways and mechanisms evidently are involved, which moreover differ from species to species.^40,44^

### Conclusions

Our results suggest that, in *Drosophila*, *Aedes*, and likely many other insect species, the strong aversive effects of catnip are largely mediated by the activation of the conserved irritant receptor TRPA1 by iridoids. Insect repellents have been traditionally thought to activate (or interfere with) insect olfactory and/or gustatory transduction cascades, but our results add to a body of evidence suggesting that plant defensive compounds also target the invertebrate irritant receptor TRPA1.^25,35,37,45^

Interestingly, despite TRPA1’s broad conservation in a variety of phyla, not all animals are repelled by catnip. As is well known, cats love catnip, and other vertebrates are not known to find catnip aversive, consistent with our observation that human TRPA1 is not activated by catnip. Indeed, not even all insects are repelled by catnip. The soybean aphid (*Aphis glycines*), for example, uses Z,E-nepetalactone as a pheromone component,^46^ and green lacewings (Neuroptera: Chrysopidae) are attracted to this compound, perhaps because their larvae feed on aphids.^47^ Aphids are indeed also well-known pests on catnip. Our results demonstrate that, even within the same insect species, TRPA1s seem to have diversified into catnip-sensitive and catnip-insensitive variants. From an evolutionary standpoint, it is interesting that—notwithstanding TRPA1’s fundamental function in nociceptive neurons—insect TRPA1 loci appear much more complex than their vertebrate counterparts, coding for a multitude of variants with different chemical sensitivities. In his 1964 paper, Eisner speculates that the *raison d’être* of the iridoids is to defend plants against phytophagous insects,^15^ an explanation that likely holds true for other natural insect repellents. It is tempting to speculate that TRPA1’s diversification in insects is the result of an evolutionary arms race between insects and chemical defensive mechanisms in plants.

Our work demonstrates how catnip iridoids function as powerful natural insect repellents because they activate the insect TRPA1, an ancient receptor for noxious and irritant chemicals—explaining at once catnip’s powerful aversive effect and its broad spectrum of insect targets. Further experiments will reveal the molecular mechanism behind the selective activation of some, but not all, TRPA1 variants. Yet the fact that catnip activates a number of insect TRPA1 variants, combined with the observation that catnip appears ineffective toward vertebrate TRPA1 (and human in particular), supports the notion that this property may be further exploited to design class- or even group-selective insect repellents.

## STAR★METHODS

### RESOURCE AVAILABILITY

The sequences reported in this paper have been deposited in GenBank with the following accession numbers: AaegTRPA1-G (GenBank: MW389509), AaegTRPA1-F (GenBank: MW389510), AaegTRPA1-C (GenBank: MW389511), and AaegTRPA1-H (GenBank: MW389512).

#### Lead contact

Further information and request for resources and reagents should be directed to and will be fulfilled by the leads contact, Marcus Stensmyr (marcus.stensmyr@biol.lu.se)

#### Materials availability

This study did not generate new unique reagents.

#### Data and code availability

This study did not generate datasets or code. Raw data are available upon request from corresponding author Marcus Stensmyr (marcus.stensmyr@biol.lu.se).

### EXPERIMENTAL MODEL AND SUBJECT DETAILS

#### Animal rearing

*Ae. aegypti* and *An. gambiae s.s* were reared and kept in an environmental room under LD 12:12 h cycle at 26–28°C, 79% RH. Eggs were hatched by adding deoxygenated water with ground fish food (#028483, Tetra, Arken Zoo, Sweden) inside a plastic container (L: 32 × W:17 × H: 10 cm). Post-hatching, larvae were fed daily with fish food. The pupae were placed in small cups with distilled water and moved to a mesh cage (L: 30 × W: 30 × H: 30 cm, DP100B, Bugdorm store, Taiwan), and allowed to eclose. Adult mosquitoes were fed on 10% sucrose solution (weight: volume in distilled water) from a cotton wick inserted into a vial. Mosquitoes were blood-fed using an artificial blood feeder (CG-1836, Chemglass Life Sciences, USA) filled with defibrinated sheep blood (#337, HåtunaLab, Sweden) (heated to 37°C), spiked with 10mM ATP (A1852, Sigma-Aldrich) for about 2 h per cage. Blood-fed mosquitoes were subsequently allowed to feed on 10% sucrose solution. *An. colluzzii* was reared similarly, except under a 14:10 h light cycle, and blood-fed on mice, according to a protocol approved by the Johns Hopkins University Animal Care and Use Committee. The AaegTRPA1^−/−^ mutant^39^ carries an insertion in the region that corresponds to exon 12 (coding for the ion channel domain region of the protein). Since this is a common exon, all transcripts from the *AaegTRPA1* locus are affected by the insertion and will prematurely terminate. All tested ant workers originated from a *Formica rufa* colony, kept in a climate chamber at 25°C, 50% relative humidity, and a LD 12 h:12 h regime at Lund university, Sweden. The colony was housed in a plastic box (L: 40 × W: 20 × H: 10 cm) connected to a foraging arena (through a silicone pipe), where foragers were allowed to freely forage for food. Male Blue Asian scorpions (*Heterometrus cyaneus*) were kept individually in a clear plastic container with small air-holes under LD 12:12h regime at 25°C, 65% RH. Each container was filled with potting soil (4–5 cm depth, https://www.plantagen.se, Sweden) and a small plastic pot (H: 6, Ø: 8 cm, https://www.plantagen.se, Sweden) for hiding. The scorpions were fed 2–3 live crickets per day. Prior to each experiment, the scorpions were food deprived for 24h.

#### *Drosophila* Cell Culture

*Drosophila* S2R+ cells were cultured in GlenClone Schneider’s Insect Medium supplemented with 10% FBS and 1% penicillin/streptomycin (100 units/mL and 100 μg/mL respectively; Fisher Scientific) and maintained in 25 cm^2^ tissue culture flasks with plug seal caps at RT. The sex of this cell line is male.

### METHOD DETAILS

#### Chemical reagents

Allyl isothiocyanate, DEET and paraffin oil was obtained from Sigma-Aldrich. A racemic mix of Z,E-nepetalactone and E,Z-nepetalactone was kindly provided by the Pheromone Group at Lund University, Sweden. Catnip oil was purchased from Biofinest (Catnip essential oil – 100% undiluted, http://biofinest.com, USA). Paraffin oil was used as solvent for all behavioral experiments.

#### Membrane-feeding assay

Non-blood fed female *Ae. aegypti* and *An. gambiae s.s* (5–10 days old) were allowed to choose between two glass jacketed membrane feeders (CG-1836, Chemglass Life Sciences, USA) prepared by stretching a thin layer of parafilm laboratory film, filled with 6mL of defibrinated sheep blood (337, Håtunalab AB, Sweden), heated to 37°C and spiked with 10mM ATP (A1852, Sigma-Aldrich). A nylon sock previously worn for 12 h by a human subject was stretched over the parafilm and tied around the feeder. The following substances were applied between the parafilm and the nylon sock: catnip 1 g (Katzenminzen, Arken Zoo, Sweden), 150 μL nepetalactone (10^−7^ diluted in paraffin oil). Females were allowed to feed for 45 min. Choice was determined by observing every fifth min the number of mosquitoes feeding on each feeder. 25–30 females were used per replicate (n = 6).

#### Constrained feeding access assay

This assay is a modification of the arm-in-cage assay, where a human hand is exposed against the mesh on the outside of the cage (L: 30 × W: 30 × H: 30 cm). 20 non-blood fed mated female *Ae. aegypti* (5 – 10 days old) were allowed to probe and “try” to feed on a human hand held at ~1 cm distance from the cage, enabling the mosquitoes to probe, but not to actually feed. The stimulus was the palm of a human hand covered with 1 mL of catnip oil (Biofinest, USA), whereas the other hand served as control. Number of mosquitoes landing on the mesh and probing were recorded after 2, 4, and 6 min. An intended biting index (BI) was calculated as follows: (#stimulus- # control) / (#stimulus+ #control) where #stimulus indicates the number of mosquitoes trying to feed on the catnip spiked hand and the #control indicates number of mosquitoes trying to feed on the hand without catnip (n = 6).

#### Uniport assay

A modified uniport assay was built based on Raji et al.^19^ to assess mosquito attraction to human host stimuli. The uniport is made of a plexiglass tube (L: 75, Ø: 13 cm) attached to a small cylindrical trap (L:10 cm, W: 5 cm), which houses the mosquitoes before the experiment. The other end of the plexiglass is a hollow box (L: 25 × W: 20 × H: 20 cm) connected to the stimulus chamber. CO_2_ enriched carbon-filtered humidified air was released through the stimulus chamber to create an airflow of 1L/min (80.PMR1–017993, Scantec Nordic, Sweden) toward the release trap. The final concentration for CO_2_ in the assay was maintained at 2500–2700 ppm by a carbon dioxide monitor (Extech CO240, Elfa, Sweden). The airflow was maintained at 9 L/m by an air flowmeter (80.PMR1–012793, Scantec Nordic, Sweden). Approximately 5 days old mated non-blood fed female *Ae. aegypti* were used for each experiment. Mosquitoes were released from the small cylinder trap and allowed to respond to stimuli (human subject arm with and without catnip) for 8 min. Mosquitoes were considered attracted if they were able to fly upwind through the tube into the attraction trap. Mosquitoes that moved out of the cylindrical trap where considered activated. A blank trial with no odor stimulus was run to ascertain that the set-up was clean. Ten females were released per repetition (n = 10).

#### Close proximity response assay

Each mosquito (*Ae. aegypti* or *An. colluzzi*) was transferred to a cage (W:30 × L: 30 × H: 30 cm, BugDorm, Taiwan) and allowed to acclimatize for 5 min before starting each experiment. A 1000 μL pipette tip containing a piece of filter paper soaked with the test stimulus was used to approach the mosquito. The pipette tip was placed on the cage wall where the mosquito was resting. The mosquito was then observed for 30 s, and the time until take-off noted. Three odorants were used: paraffin oil, 100% DEET (Sigma-Aldrich), and catnip oil (Biofinest, USA). Exposure was randomized, and the mosquito given 2 min to rest between each exposure (n = 30).

#### Scorpion behavioral assay

All experiments were carried out under infrared light. One Asian blue forest scorpion per assay was used (n = 4). Each scorpion was released in the middle of an arena (Ø = 150 cm). The inside of two pots were lined with filter paper (one of which scented with catnip oil and the other without) and served a hiding places for the scorpions. The pots were positioned at opposite sides. The scorpion was given 10 min to choose a hiding place.

#### Ant behavioral assay

A glass Petri dish was used for each assay (Ø = 10 cm). One worker ant was used per replicate (n = 20). The crickets were sacrificed right before each repetition. An aliquot of 5 μL of catnip oil (Biofinest, USA) was added onto one of the crickets. Each ant was given 4 min to approach the crickets. The Petri dish was cleaned with 70% ethanol after each replicate and allowed to air dry before next replicate. The placement of the crickets was random for each replicate. The ants’ movements were tracked using Noldus Ethovision XT (Noldus, the Netherlands).

#### *Drosophila* oviposition assay

Oviposition assays were conducted following Manourian et al.^16^ 20 mated females were introduced to two-choice Petri dishes. The two-choice dishes were made by diving a 47 mm Petri dish into two halves. One half was treated with 0.05 g catnip or 150 μL nepetalactone (10^−6^), and the other half as a control (fly food mixed with water). After 24 h, the #eggs in each side was counted and an Oviposition Index (OI) was calculated (OI = #eggs in treatment − #eggs in control) / (Total number of eggs).

#### Cloning of Ae. aegypti TRPA1 full-length coding sequences

Predicted transcript from *Ae. aegypti* TRPA locus (AAEL001268) were assembled from VectorBase (https://vectorbase.org/vectorbase/app/). *Ae. aegypti* TRPA1 cDNAs for each isoform were isolated from total RNA from newly hatched *Ae. aegypti* (black eye Liverpool, purchased from the BEI Resources repository) by retrotranscription using Superscript III reverse transcription (Life Technologies) using gene-specific primers (see Key resources table) followed by PCR. The gene specific primers for the 5′ end included the Kozak sequence CAAAAC just upstream of the ATG. All cDNAs were cloned into pCR8/GW/TOPO® TA (Invitrogen) and sequenced before being transferred into pAC-GW expression vector for *in vitro* expression. Note that our nomenclature follows vectorbase, and that our AaegTRPA1-C/K/I and AaegTRPA1-G correspond to variants A and C of reference,^35^ respectively.

#### Cell transfection

The construction of pAC-GFP, pAC-dTRPA1-A and pAC-dTRPA1-C was described before.^32^ These vectors and the vectors containing the TRPA1 isoforms from *Ae. aegypti* (see above) were transfected into S2R^+^ cells. 0.5 μg of the pAC-GFP vector and 1.5 μg of either one of the vectors harboring TRPA1 from *Drosophila* or from *Aedes* were incubated with 4 μL of transfection reagent and 200 μL of transfection buffer (jetPRIME, PolyplusTransfection) for 10 min and then added to S2R^+^ cells grown on glass coverslips. Transfected cells were incubated at RT for at least 36 hours to allow for gene expression.

#### Electrophysiology

Whole-cell voltage-clamp recording was performed on S2R^+^ transfected cells identified by GFP fluorescence. The intracellular solution contained 140 mM K-Gluconate, 2 mM MgCl_2_, 0.01 mM CaCl_2,_ 1 mM EGTA, 5 mM HEPES, and 1 mM Na_2_ATP; pH was adjusted to 7.2 ± 3 and the osmolarity was adjusted to 315 ± 5 mOsml with sucrose, and was stored at −20°C. The extracellular solution contained 140 mM NaCl, 5 mM KCl, 1 mM CaCl_2_, 1 mM HEPES, and 10 mM glucose; pH was adjusted to ~7.2 with NaOH and the osmolarity was adjusted to 305 ± 5 mOsml with sucrose. The resistance of patch pipettes ranged from 3 to 7 MΩ. Recordings were obtained with an AxoPatch 200B amplifier (Axon Instruments), analyzed with AxoGraph software, scaled to 1 × output gain, lowpass filtered with 5KHz, and digitized with a Digidata 1320A. Bath offset and capacitance were compensated; series resistance was 9.5 ± 5.5 MΩ without compensation. Recordings were made at RT and heating was achieved by using an inline heater (HPT-2A, ALA Scientific Instruments), TC-20 temperature controller (NPI Electronics), and a T-384 thermocouple (Physitemp Instruments) tethered to the electrode holder, such that the tip of the thermocouple was at a close distance to the electrode. Stimulation via chemicals was achieved by bath perfusion of extracellular solution containing 100 mM allyl isothiocyanate (AITC, MilliporeSigma), 100 μM nepetalactone (Toronto Research Chemicals), and 0.1%–0.5% catnip essential oil (Biofinest or Aromatics International) diluted in 1% DMSO:EtOH (1:1). Cells were held at constant −60 mV and currents were monitored during heat and chemical stimulation.

### QUANTIFICATION AND STATISTICAL ANALYSIS

Boxplots in Figures 1 and 3 are as follows; thick lines mark the medians, box are the first and third quartiles, and whiskers indicate interquartile range from the 25^th^-75^th^ percentiles. All statistics were performed using R (https://cran.r-project.org/). Statistical details related to sample size and p values are reported in the figure legends, with a star denoting p < 0.05. Current density measurements in Figure 2 were obtained by normalizing peak current amplitude with cell capacitance. Boxplots were generated in MATLAB®. In the boxplots, thick lines mark the medians, bottom and top lines indicate the first and third quartiles, and whiskers represent data range. Data values beyond the whiskers are outliers and plotted as points. In Figure 2D, the number of cells used for each treatment (n) is as follows: AITC (n = 5), solvent (n = 5), 0.1% Catnip (n = 4), 100 μM Nepetalactone (n = 5). In Figure 2H the number of cells used for each treatment (n) is as follows: heat (n = 7), AITC (n = 5), solvent (n = 7), 0.1% catnip (n = 5), 0.2% catnip (n = 6), 0.5% catnip (n = 5), nepetalactone (n = 3), mock (n = 5).

## Supplementary Material

1

## Figures and Tables

**Table:** KEY RESOURCES TABLE

REAGENT or RESOURCE	SOURCE	IDENTIFIER
Bacterial and virus strains		
*E. coli*	New England Biolabs	C3019I
Biological samples		
Total RNA from *A. aegypti* black eye liverpool newly hatched	BEI Resources	Cat# NR-42506
Chemicals, peptides, and recombinant proteins		
Defibrinated sheep blood	HåtunaLab, Sweden	#337
Allyl isothiocyanate	Sigma	Cat#377430-100G
Nepetalactone	Toronto Research Chemicals	Cat# N390055
Racemic nepetalactone	Pheromone Group, Lund University Sweden	N/A
Catnip essential oil	Biofinest	N/A
Catnip (dried)	Arken Zoo, Sweden	N/A
Adenosine 5′-triphosphate (ATP) disodium salt hydrate	Sigma-Aldrich	Cat# A1852
D-(+)-Sucrose octaacetate	Sigma-Aldrich	Cat# 252603
Penicilin/streptomycin mixture	Fisher Scientific	Cat# BP2959-50
Fetal Bovine Serum	Sigma	Cat# F2442-500ML
Tretra Tabimin Fishfood	Arken Zoo, Sweden	028483
Schneider’s *Drosophila* Medium	GIBCO	Cat#21-720-024
Paraffin oil	Sigma-Aldrich	Cat# 18512
Critical commercial assays		
ZymoPUREPlasmid MiniprepKit	Zymo Research	Cat#D4212
iScript Select cDNA Synthesis Kit	BioRad	Cat# 1708896
pCR8/GW/TOPO TA Cloning Kit	Thermo Fisher Scientific	Cat#K250020
Gateway LR Clonase II Enzyme mix	Thermo Fisher Scientific	Cat#11791020
jetPRIME® DNA and siRNA Transfection Reagent	Genesee Scientific Corp	Cat# 55-132
Deposited data		
*Aaeg*TRPA1-C cDNA		GenBank: MW389511
*Aaeg*TRPA1-F cDNA		GenBank: MW389510
*Aaeg*TRPA1-G cDNA		GenBank: MW389509
*Aaeg*TRPA1-H cDNA		GenBank: MW389512
Experimental models: cell lines		
*Drosophila*: S2R+ cells	Gift from the Carthew lab	CVCL_Z831
Experimental models: organisms/strains		
*Ae. aegypti / Orlando^wt^*	DeGennaro Lab, Florida International University, Miami, USA	N/A
*Ae. aegypti / Orco^5/16^*	DeGennaro Lab, Florida International University, Miami, USA	N/A
*Ae. aegypti* / TRPA1	Vosshall lab, The Rockefeller University, New-York, USA	N/A
*An. gambiae / Keele*	Noushin Emami Lab, Stockholm University, Sweden	N/A
*Formica rufa*	Dacke Lab, Lund University, Sweden	N/A
*Drosophila melanogaster^w1118^*	Stensmyr Lab, Lund University, Sweden	N/A
*D. melanogaster^trpa1^*	Bloomington stock center	26504
*Heterometrus cyaneus*	Dacke Lab, Lund University	N/A
Oligonucleotides		
*Aaeg* TRPA1 E,G,H cDNA F 5-caaaacATGCCAACTCCGCTCTATCTG-3′	This study	N/A
*Aaeg* TRPA1 I,K cDNA F 5′-aaaacATGTTACCAATTATGATGTACAG-3’	This study	N/A
*Aaeg* TRPA1 F cDNA F 5′- caaaacATGCTCATCTTCAATGAAGTT-3’	This study	N/A
*Aaeg* TRPA1 cDNA R 5′- CTACTTGCTAATCGACTTGTTAAAAC-3′	This study	N/A
Recombinant DNA		
pAC: GFP	^32^	N/A
pAC: dTRPA1-A	^32^	N/A
pAC: dTRPA1-C	^32^	N/A
pAC: *Aaeg*TRPA1-C	This study	N/A
pAC: *Aaeg*TRPA1-F	This study	N/A
pAC: *Aaeg*TRPA1-G	This study	N/A
pAC: *Aaeg*TRPA1-H	This study	N/A
Software and algorithms		
AxoGraph X Software (Version 1.5.4)	Axon Instruments	https://axograph.com
Geneious*®*	Geneious	https://www.geneious.com
VectorBase		https://vectorbase.org/vectorbase/app
MATLAB*®*	MathWorks	https://www.mathworks.com/products/matlab.html
EthoVision XT	Noldus	https://www.noldus.com
Illustrator CC 21.02	Adobe	https://www.adobe.com/
R	R core team 2013	https://cran.r-project.org
Other		
Bugdorm Cages (30×30×30)	BugDorm Store	https://shop.bugdorm.com
Mosquito feeders (50mm)	Chemglass Life Sciences, USA	CG-1836
CO_2_ flowmeter	Scantec Nordic, Sweden	80.PMR1-017993
Air flowmeter	Scantec Nordic, Sweden	80.PMR1-012793
CO_2_ monitor	Elfa Distrelec, Sweden	1501-8116-7371-200
Whatman Filter Paper	Sigma-Aldrich	1001500
Uniport olfactometer	Custom-made	N/A

## Highlights

- Catnip is a broad-acting insect repellent
- The repellency is mediated via iridoids such as nepetalactone
- Catnip and nepetalactone activate select insect TRPA1 isoforms
- TRPA1-deficient mosquitoes show no aversion to catnip
