# Supplementary material for: The irritant receptor TRPA1 mediates the mosquito repellent effect of catnip
Source: Curr Biol. Author manuscript; Available in PMC 2022 May 10. (PMC8764911; doi:10.1016/j.cub.2021.02.010)
Supplement: 1 [file NIHMS1769779-supplement-1.pdf]

Current Biology, Volume *31*

## Supplemental Information

# The irritant receptor TRPA1 mediates the mosquito repellent effect of catnip

**Nadia Melo, Matthew Capek, Oscar M. Arenas, Ali Afify, Ayse Yilmaz, Christopher J. Potter, Peter J. Laminette, Alessia Para, Marco Gallio, and Marcus C. Stensmyr**

| **Family / species** | **Effect** | **Reference** |
|---|---|---|
| **Arachnida** | | |
| Ixodida (ticks) | | |
| *Rhipicephalus* (hard ticks) | SR | S1 |
| *Dermanyssus* (poultry mites) | SR | S1 |
| *Dermatophagoides* (dust mites) | SR | S2 |
| **Insecta (insects)** | | |
| Blattodea (cockroaches/termintes) | | |
| *Blatella* (German cockroaches) | SR | S3 |
| *Periplaneta* (American cockroaches) | SR | S4 |
| *Isoptera* (termites) | SR, CR | S5 |
| *Reticulitermes* (termites) | SR, CR | S5 |
| Hemiptera (true bugs) | | |
| *Acanalonia* (planthopper) | SR | S6 |
| *Cercopidae* (froghoppers) | SR | S6 |
| *Sitobion* (grain aphids) | SR | S1 |
| *Cimex lectularius* (bed bug) | SR | S7 |
| *Aphis* (aphids) | SR | S8 |
| Hymenoptera (wasps/bees/ants) | | |
| *Camponotus* (carpenter ants) | SR | S6 |
| *Pogonomyrmex* (harvester ants) | SR | S6 |
| *Vespula* (social wasps) | SR | S9 |
| *Polistes* (paper wasps) | SR | S9 |
| Coleoptera (beetles) | | |
| *Coptotomus* (diving beetles) | SR | S6 |
| *Photinus* (fireflies) | SR | S6 |
| *Cicindela* (tiger beetles) | SR | S6 |
| *Ataenius* (dung beetles) | SR | S6 |
| *Disonycha* (flea beetles) | SR | S6 |
| *Callosobruchus* (bean beetles) | SR | S10 |
| *Tribolium* (flour beetles) | SR | S11 |
| *Leichenum* (darkling beetles) | SR | S6 |
| *Hymenorus* (comb-clawed beetles) | SR | S6 |
| *Harmonia* (lady beetles) | SR | S12 |
| *Derelomus* (flower weevil) | SR | S6 |
| *Conotrachelus (tree weevil)* | SR | S6 |
| Curculionidae sp (snout beetles) | SR | S6 |
| Staphylinidae sp (rove beetles) | SR | S6 |
| Trichoptera (caddisflies) | | |
| *Nectopsyche* (white millers) | SR | S6 |
| *Oecetis* (long-horned caddisflies) | SR | S6 |
| Lepidoptera (butterflies/moths) | | |
| *Pieris* (garden whites) | SR | S13 |
| Diptera (true flies) | | |
| *Stomoxys* (stable flies) | SR, FD, OR | S14, S15 |
| *Haematobia* (horn flies) | SR, FD | S16 |
| *Musca* (house flies) | SR | S4 |
| *Aedes* (mosquitoes) | SR, CR, BD, T | S17-S27 |
| *Culex* (mosquitoes) | SR, CR | S17, S18, S27 |
| *Anopheles* (mosquitoes) | SR, T | S17-S19 |
| *Simulium* (black flies) | SR | S27 |

**Table S1. Arthropods repelled by catnip and/or nepetalactone. Related to Figure 1.**
SR: spatial repellent; CR: contact repellent; FR: feeding repellent; OR: oviposition repellent; BD: biting deterrent; T: toxic. See supplemental references.

## Supplemental references


S1. Birkett, M.A., Hassanalo, A., Hoglund, S., Petterson, J., and Pickett, J.A. (2011). Repellent activity of catmint, *Nepeta cataria,* and iridoid nepetalactone isomers against Afro-tropical mosquitoes, ixodid ticks and red poultry mites. Phytochem. *72*, 109-114.

S2. Khan, M.A., Jones, I., Loza-Reyes, E., Cameron, M.M., Pickett, J.A., Birkett, M.A. (2012). Interference in foraging behavior of European and American house dust motes *Dermatophagoides pteronyssinus* and *Dermatophagoides farinae* (Acari: Pyroglyphidae) by catmint, *Nepeta cataria* (Lamiaceae). Exp. Appl. Acarol. *57,* 65-74.

S3. Peterson, C.J., Nemetz, L.T., Jones, L.M., and Coats, J.R. (2002). Behavioral activity of catnip (Lamiaceae) essential oil components to the german cockroach (Blattodea: Blattellidae). J. Econ. Entomol. *95,* 377-380.

S4. Schultz, G., Simbro, E., Belden, J., Zhu, J., and Coats, J. (2004). Catnip, *Nepeta cataria* (Lamiales: Lamiaceae) – A closer look: seasonal occurrence of nepetalactone isomers and comparative repellency of three terpenoids to insects. Environ. Entomol. *33,* 1562-1569.

S5. Peterson, C.J and Ems-Wilson, J. (2003). Catnip essential oil as a barrier to subterranean termites (Isoptera: Rhinotermitidae) in the laboratory. J. Econ. Entomol. *96,* 1275-1282.

S6. Eisner, T. (1964). Catnip: Its raison d'Etre. Science. *146,* 1318-1320.

S7. Shi, X., Wang, C., Simon, J.E., Reichert, W., and Wu, Q. (2020). Repellency of novel catnip oils against the bed bug (Hemiptera: Cimicidae). J. Med. Entomol. doi: 10.1093/jme/tjaa218

S8. Birkett, M.A., Bruce, T.J.A., and Picket, J.A. (2010). Repellent activity of *Nepeta grandiflora* and *Nepeta clarkei* (Lamiaceae) against the cereal aphid, *Sitobion avenae* (Homoptera: Aphididae). Phytochem. Lett. *3,* 139-142.

S9. Zhang, Q-H., Schneidmiller, R.G., amd Hoover, D.R. (2013). Essential oils and their compositions as spatial repellents for pestiferous social wasps. Pest. Manag. Sci. *69,* 542-552.

S10. Sabbour, M.M.A. (2019). Efficacy of natural oils against the biological activity on *Callosobruchus maculatus* and *Callosobruchus chinensis* (Coleoptera: Tenebrionidae). Bull. Natl. Res. Cent. *43,* 1-8.

S11. Arthur, F.H., Fontenot, E.A., and Campbell, J.F. (2010). Evaluation of catmint oil and hydrogenated catmint oil as repellents for the flour beetles, *Tribolium castaneum* and *Tribolium confusum.* J. Insect Sci. *11,* 1536-2442.

S12. Riddick, E.W., Brown A.E., and Chauhan, K.R. (2008). *Harmonia axyridis* adults avoid catnip and grapefruit-derived terpenoids in laboratory bioassays. Bull. Insectol. *61,* 81-90.

S13. Li-Min, W., Wen-Yan, LU., Guo-Hui, Y., Yang-Ling, XU., Xian-Ru, G., and Mei-Hao, L. (2010). Repellent effect of non-host vegetables and their extracts on *Pieris rapae* L. Chin. J. Eco-Agric.. *18,* 1311-1316.

S14. Zhu, J.J., Li, A.Y., Pritchard, S., Tangtrakulwanich, K., Baxendale F.P., and Brewer, G. (2011). Contact and fumigant toxicity of a botanical-based feeding deterrent of the stable fly, *Stomoxys calcitrans* (Diptera: Muscidae). J. Agric. Food. Chem. *59,* 10394-10400.

S15. Zhu, J.J. Dunlap, C.A., Behle, R.W., Berkebile, D.R., and Wienhold, B. (2010). Repellency of a wax-based catnip-oil formulation against stable flies. J. Agric. Food. Chem. *58,* 12320-12326.

S16. Zhu, J.J., Brewer, G.J., Boxler, D.J., Friesen, K., and Taylor, D. B. (2015). Comparisons of antifeedancy and spatial repellency of three natural product repellents against horn flies, *Haematobia irritans* (Diptera: Muscidae). Pest Manag. Sci. *71,* 1553-1560.

S17. Amer, A., and Mehlhorn, H. (2006). Repellenct effect of forty-one essential oils against *Aedes, Anopheles,* and *Culex* mosquitoes. Parasitol. Res. *99,* 478-490.

S18. Doraysamy., Mulyaningsih, B., and Ernaningsih. (2016). Repellent activity of catnip extract (*Nepeta cataria L,*) against *Aedes aegypti* mosquito as dengue vector. TMJ. *2,* 93-102.

S19. Chauhan, K.R., Klun, J.A., Debboun, M., and Kramer, M. (2005). Feeding deterrent effects of catnip oil components compared with two synthetic amides against *Aedes aegypti.* J. Med. Entomol. *42,* 643-646.

S20. Uniyal, A., Tikar, S.N., Mendki M.J., Singh, R., Shukla, S., Agrawal, O.P., Veer, V., and Sukumaran, D. (2016). Behavioral Response of *Aedes aegypti* mosquito towards essential oils using olfactometer. J. Arthropod-Borne Dis. *10,* 370-380.

S21. Obermayr, U., Ruther, J., Bernier, U.R., Rose, A., and Geier, M. (2015). Evaluation of a Push-Pull approach for *Aedes aegypti* (L.) using a novel dispensing system for spatial repellents in the laboratory and in a semi-field environment. Plos One. *10, DOI:10.1371/journal.pone.0129878*

S22. Peterson, Christopher J., and Joel R. Coats. "Catnip essential oil and its nepetalactone isomers as repellents for mosquitoes." Recent Developments in Invertebrate Repellents. Am. Chem. Soc. 2011. 59-65.

S23. Polsomboon, S., Grieco, J.P., Achee, N.L., Chauhan, K.R., Tanasinchayakul, S., Pothikasikorn, J., and Chareonviriyaphap, T. (2008). Behaviorak responses of catnip (*Nepeta cataria*) by two species of mosquitoes, *Aedes aegypti* and *Anopheles Harrisoni,* in Thailand. J. Am. Mosq. Con. Assoc. *24,* 513-519.

S24. Ali, A., Tabanca, N., Demirci, B., Blythe, E.K., Baser, K.H.C., and Khan, I.A. (2016). Chemical composition and Biological activity of essential oils from four *Nepeta* species and hybrids against *Aedes aegypti* (L.) (Diptera: Culicidae). Rec. Nat. Prod. *10,* 137-147.

S25. Sathrantriphop, S., White, S.A., Achee, N.L., Sanguanpong, U., and Chareonviriyaphao, T. (2014). Behavioral responses of *Aedes aggypti, Aedes albopictus, Culex quinquefasciatus,* and *Anopheles minimus* against various synthetic and natural repellent compounds. J. Vec. Ecol. *39,* 328-339.

S26. Sathrantriphop, S., Achee, N.L., Sanguanpong, U., and Chareonviriyaphap, T. (2015). The effects of plant essential oils on escape response and mortality rate of *Aedes aegypti* and *Anopheles minimus.* J. Vec. Ecol. *40,* 318-326.

S27. Spero, N.C., Gonzalez, Y.I., Scialdone, M.A., and Hallahan, D.L. (2008). Repellency of hydrogenated catmint oil formulations to black flies and mosquitoes in the field. J. Med. Entomol. *45,* 1080-1086.

S28. Webb, C.E., and Russell, R.C. (2007). Is the extract from the plant catmint (*Nepeta cataria*) repellent to mosquitoes in Australia? J. Amer. Mosq. Con. Assoc. *23,* 351-354.
